# Supplementary material for: Epilepsy-linked kinase CDKL5 phosphorylates voltage-gated calcium channel Cav2.3, altering inactivation kinetics and neuronal excitability
Source: Nat Commun. 2023 Dec 11;14:7830. doi: 10.1038/s41467-023-43475-w (PMC10713615; doi:10.1038/s41467-023-43475-w)
Supplement: Supplementary file 1 — Supplementary Information [file 41467_2023_43475_MOESM1_ESM.pdf]

Supplementary Information

Epilepsy-linked kinase CDKL5 phosphorylates voltage-gated calcium channel Cav2.3, altering inactivation kinetics and neuronal excitability

Marisol Sampedro-Castañeda[1,5,*], Lucas L. Baltussen[1,4,5], André T. Lopes[1], Yichen Qiu[2], Liina Sirvio[1], Simeon R. Mihaylov[1], Suzanne Claxton[1], Jill C. Richardson[3], Gabriele Lignani[2], Sila K. Ultanir[1]*

1. Kinases and Brain Development Lab, The Francis Crick Institute, 1 Midland Road, London NW1 1AT, United Kingdom

2. Department of Clinical and Experimental Epilepsy, UCL Queen Square Institute of Neurology, Queen Square House, London WC1N 3BG, United Kingdom

3. Neuroscience, MSD Research Laboratories, 120 Moorgate, London, EC2M 6UR, United Kingdom

* Correspondence: sila.ultanir@crick.ac.uk, marisol.sampedro-castaneda@crick.ac.uk

4. Current address: Laboratory for the Research of Neurodegenerative Diseases (VIB-KU Leuven), Department of Neurosciences, ON5 Herestraat 49, 3000 Leuven, Belgium

5. These authors contributed equally

Supplementary Table 1. Clinical overlap between CACNA1E and CDKL5 neurodevelopmental disorders.

Clinical manifestation	CACNA1E variants[a]	CDKL5 deficiency[b]
Early onset seizures (frequently intractable)	✓	✓
Global developmental delay (including non-verbal and non-ambulatory)	✓	✓
Intellectual disabilities	✓	✓
Autistic behaviours	✓	✓
Hypotonia	✓	✓
Hyperkinetic movements	✓	✓
Abnormal sleep (EEG or narrative)	✓	✓
Visual impairment	✓	✓
Sensory symptoms	✓	✓
Motor stereotypies	✓	✓
Dystonia	✓	
Congenital contractures	✓	
Macrocephaly; atrophy	✓	
Early death	✓	
Microcephaly; atrophy		✓
Gastrointestinal symptoms		✓
Dysphagia		✓
Autonomic and breathing disturbances		✓

a. [1, 2, 3]

b. [4]

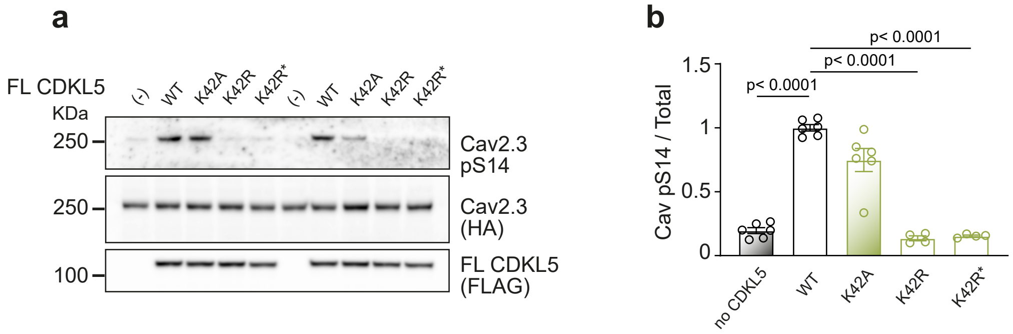

Supplementary Fig. 1: Full-length CDKL5 phosphorylates Cav2.3. a Western blot of HEK293 cells stably expressing human β3/α2δ1 subunits and transiently co-transfected with human WT HA- α1E (Cav2.3) and FLAG-CDKL5 full length (FL CDKL5: WT, K42A, K42R, K42R*+D153A kinase dead mutants). (-) transfection condition excludes CDKL5. The K42A mutation in FL-CDKL5 seems to retain some kinase activity on α1E, while K42R and K42R* mutants are fully inactive and were thus selected for electrophysiology experiments. Example blots derive from two gels run and processed in parallel. **b** Quantification of relative phospho-α1E (pS14 Cav2.3) levels for the experiment in **a** (Brown-Forsythe & Welch ANOVA, Dunnet's test; no CDKL5/WT/K42R n=6, 3 transfections, 2 technical replicates, K42R/K42R* n=4,3 transfections, 1-2 technical replicates). Data is presented as mean ± S.E.M. All source data is provided in a Source Data File.

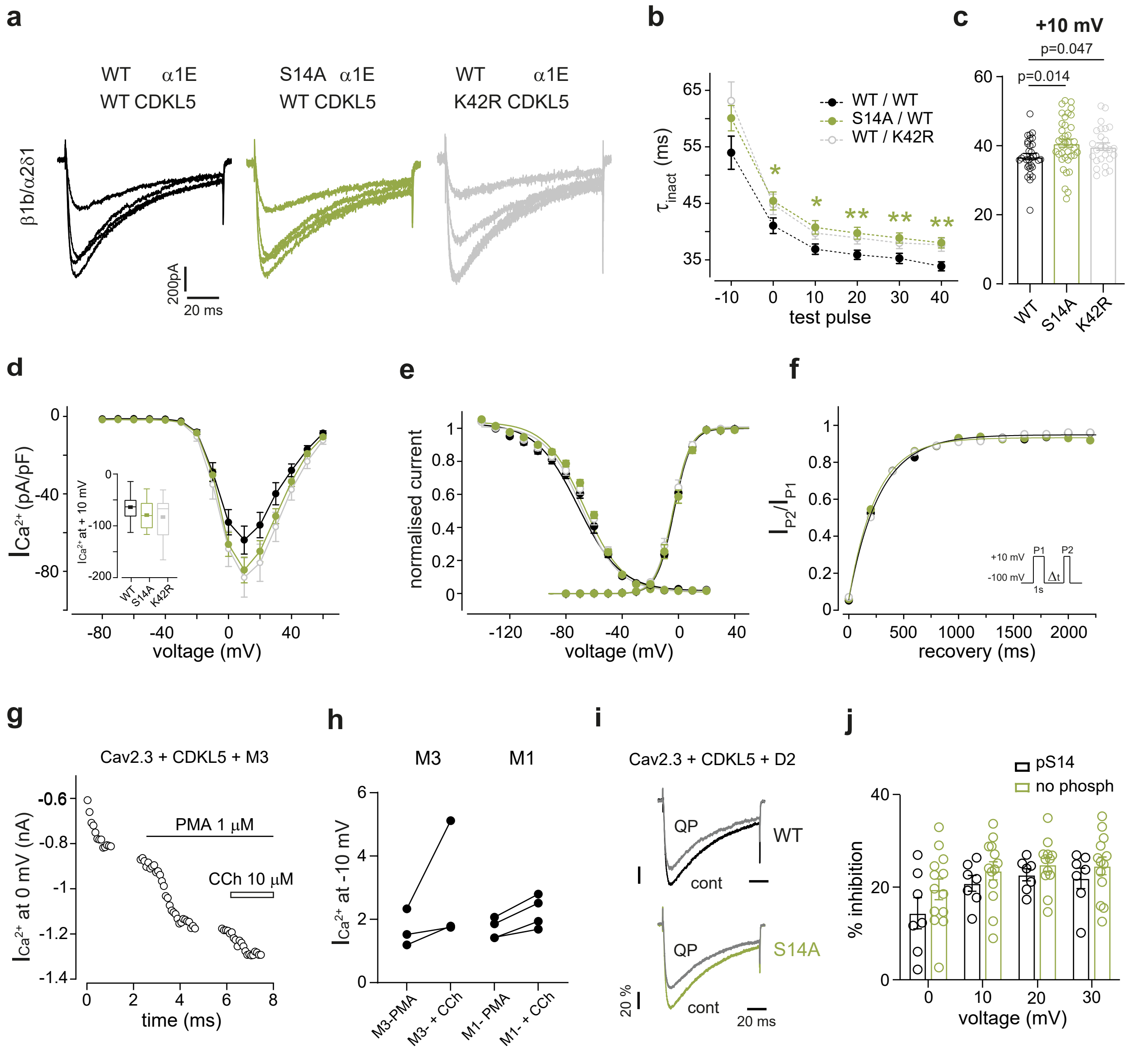

Supplementary Fig. 2: Functional characterization of phospho-Ser14 Cav2.3 in HEK 293 cells expressing β1 accessory subunit. a Depolarization-evoked current responses in HEK293 cells stably expressing human β1/α2δ1 subunits and co-transfected with human Cav2.3 (WT α1E or S14A α1E mutant) and FLAG-CDKL5 full length (WT CDKL5 or K42R*+D153A kinase dead CDKL5 mutant). Colours denote different construct combinations. Traces show steps from -10 to +20mV from -100mV; Ca^{2+} was charge carrier. **b** Open channel inactivation tau (τ_{inact}) for Cav2.3 with (WT/WT, n=28-32) and without (S14A/WT, n=34-37; WT/K45R, n=25-26) CDKL5 phosphorylation. WT/WT vs. S14A/WT: *p<0.05, **p<0.01 as indicated; WT/WT vs. WT/K42R*, p<0.01 at +40mV, p<0.05 at -10,10,20,30mV, Two-Way ANOVA, Fisher's LSD. Some of the cells included in this plot co-expressed M3 receptors; this was *per se* not found to affect inactivation kinetics. **c** Individual data points for τ_{inact} at +10mV (two-tailed unpaired t tests). **d** Current-voltage relationship for experiments in **a** (WT/WT, n=15; S14A/WT n=15-17; WT/K42R* n=12-15) and current density at +10mV (inset, p>0.05, OneWay ANOVA). Data was acquired with 100ms

voltage steps from -100mV in +10mV increments every 10s. **e** Normalised Cav2.3 conductance and voltage dependence of inactivation for the same three transfection conditions. Activation $V_{1/2}$, n: WT/WT -4±1 mV, 15; S14A/WT -3±1 mV,17; WT/K42R* -2±1 mV, 14; Inactivation $V_{1/2}$, n: WT/WT -66±2 mV,14; S14A/WT -63±1 mV,14; WT/K42R* -65±2 mV,11 ($p>0.05$, Brown-Forsythe and Welch ANOVA and One-Way ANOVA, respectively). For inactivation protocol see Methods. Solid lines are Boltzman fits to the average data. **f** Inactivation recovery time using a double pulse protocol with variable inter-pulse recovery time (inset). Recovery tau, n: WT/WT 265±21 ms, 4; S14A/WT 263±21 ms, 5; WT/K42R* 290±15 ms, 4, $p>0.05$ Kruskall-Wallis, Dunn's test). **g** Time course of Cav2.3 current amplitude at 0mV in the $\beta1/\alpha2\delta1$-stable cell line co-transfected with α1E WT, CDKL5 and muscarinic receptor type 3 (M3). PKC activation with PMA did not occlude CCh enhancing effect. **h** Relative change in current amplitude for same experiment as **g** with either M1 or M3 receptors. No muscarinic inhibition was observed. **i** Cav2.3 current at +10mV in $\beta1/\alpha2\delta1$ cell line co-transfected with α1E (WT or S14A), CDKL5 (WT) and dopamine receptor type 2 (GFP-D2). Quinpirole 100nM (QP) application resulted in reliable and reversible inhibition of WT and S14A currents. **j** Quinpirole inhibition of Cav2.3 with (n=7) or without pS14 (n=12-13) ($p>0.05$ Two-Way ANOVA, Fisher's LSD). Data for α1E S14A phosphomutant and K42R* CDKL5 conditions (no phosphorylation) were pooled together. Data is presented as mean ± S.E.M or in box plots representing minimum, maximum, median, 25/75 percentile and mean (indicated by a marker). Source data are provided as a Source Data file.

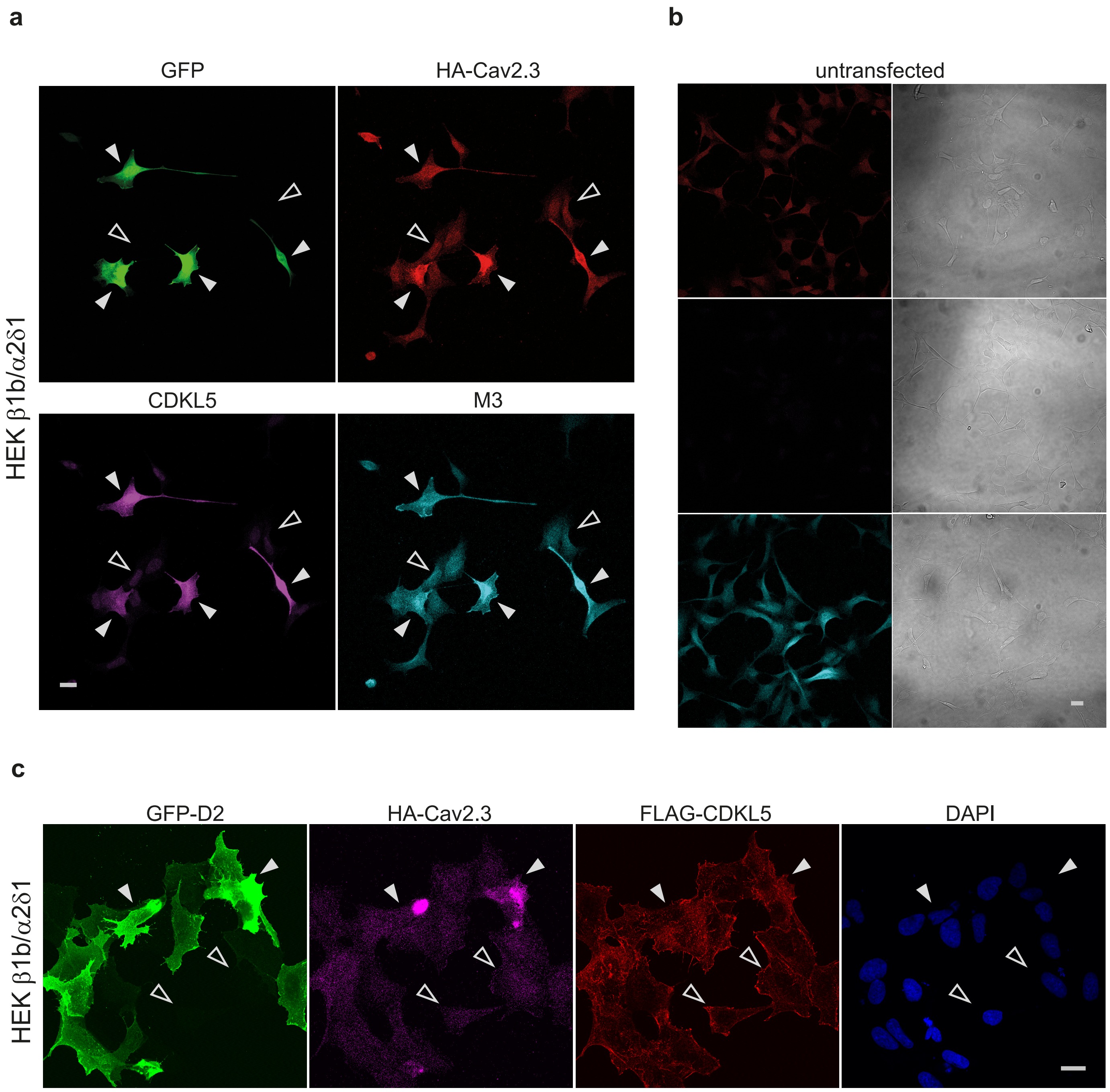

Supplementary Fig. 3: Immunocytochemical detection of co-expressed proteins in HEK293 cells. a Representative confocal image projection of HEK $\beta 1/\alpha 2\delta 1$ cells transfected with GFP, FLAG-M3, WT full-length FLAG-CDKL5 and WT HA-α1E. Coverslips are immunostained with anti-GFP (green), anti-M3 (cyan), anti-CDKL5 (magenta) and anti-HA (red). The image shows that all GFP-positive cells (filled arrows) express M3 receptor, CDKL5 and Cav2.3. GFP-negative cells (open arrows) also express these plasmids to some extent, likely owing to the higher concentrations of these plasmids in the transfection mixture. **b** Control immunostainings of untransfected cells show low background levels of staining. The background fluorescence observed in M3 stained untransfected cells may be due to endogenous levels of M3 expression

in these cells [5]. **c** Example confocal image of HEK β1/α2δ1 cells transfected with GFP-D2, WT full-length FLAG-CDKL5 and WT HA-α1E. Coverslips are immunostained with anti-GFP (green), anti-FLAG (red), anti-HA (magenta) and DAPI (blue). The pictures show that all GFP-positive (filled arrows) cells express channel and kinase. GFP-negative cells also express kinase and channel due to the high level of these plasmids in the transfection mixture. a-c data were repeated in two independent transfections in successive HEK cells passages, scale bar 20 μm. See Methods for antibody details. Note that in these experiment, low levels of GFP expression are amplified. Lower expressing cells may not be visible under the patch microscope and thus not selected for recordings.

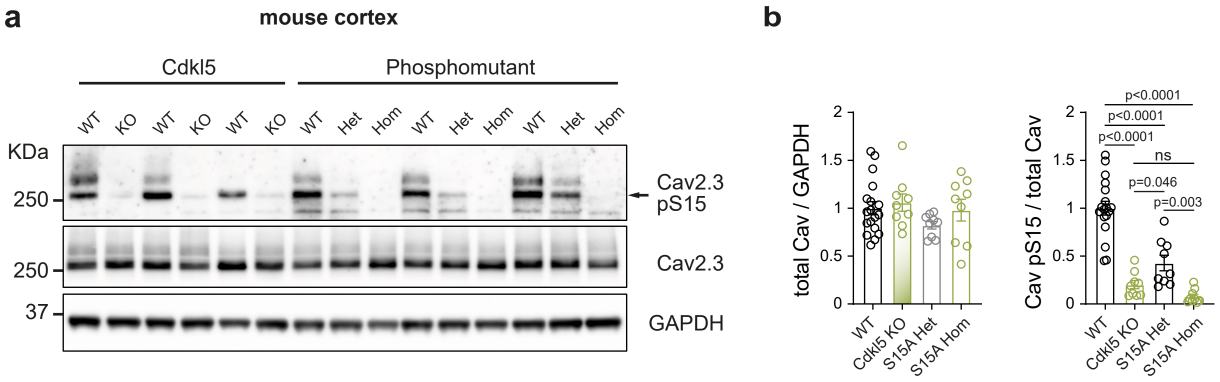

Supplementary Fig. 4: Phospho-Cav2.3 levels in Cdkl5 KO mice and Hom S15A phosphomutants are comparable. a Representative Western Blot of cortical brain lysates from 5–6-week-old Cdkl5 and phosphomutant male mice. Examples derive from two gels run and processed in parallel. **b** Corresponding quantification of total Cav2.3 (total Cav, $p>0.05$) and phospho Cav2.3 (One Way ANOVA). Data derives from 3/4 technical replicates: WT n=18 (6 mice), KO//Het//Hom n=9 (3 mice each). Band intensity was normalized first to its corresponding control (as indicated in the y axis) and then to the average of the WT signal within each blot. Part of the data was used in Fig. 3a. Data is presented as mean ± S.E.M. Source data are provided as a Source Data file.

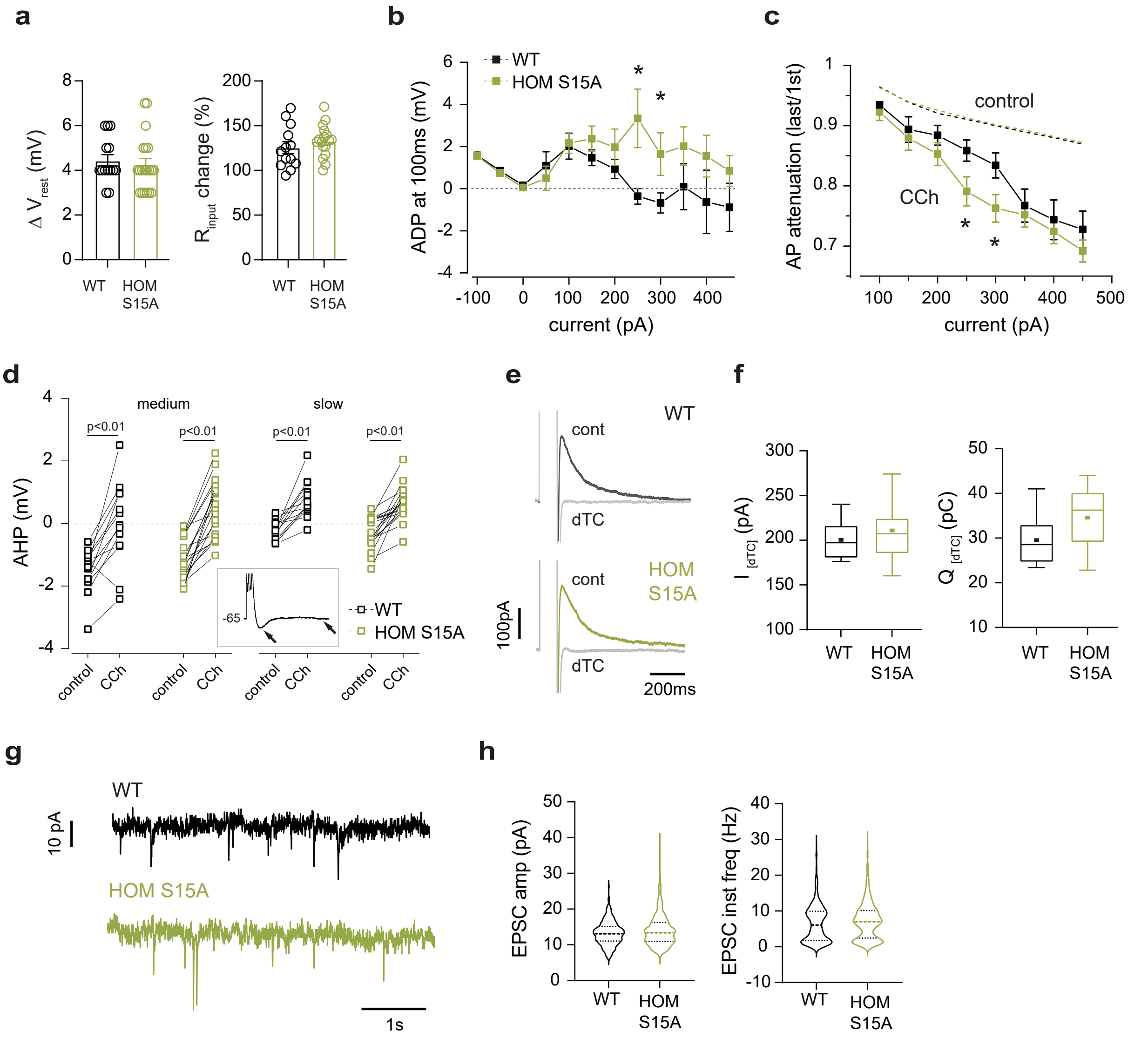

Supplementary Fig. 5: Additional CA1 neuron responses to carbachol and other intrinsic properties in adult WT and HOM S15A mice. **a** Relative change in membrane potential (V_{rest}) and resistance (R_{input}) upon 10 µM CCh application in WT (13) and HOM S15A (20 + 17 respectively) neurons (7 mice/genotype, p>0.05, unpaired t tests). **b** CCh-evoked medium duration afterdepolarization (ADP) measured 100ms post 1s-long stimulus (WT n=13, HOM n=21; p=0.07 Two-Way ANOVA; *p<0.05 Fisher's LSD). **c** Spike attenuation in control (dotted lines) and upon CCh-induced depolarization (markers) shown as ratio between last and first spike amplitude in 1s train (WT n=13, HOM n=19, Two-Way ANOVA; *p<0.05 Fisher's LSD). **d** Medium and slow duration afterhyperpolarizations (AHPs) quantified at the peak and 500ms post stimulus, respectively (inset). AHPs evoked at -65mV by a 100Hz burst of somatic current injections were equal in WT (13) and HOM S15A (18-19) mice (mAHP, -1.5 *vs* -1.2mV; sAHP, -0.2 *vs* -0.3mV, p>0.05, unpaired t test). CCh suppression of burst evoked AHPs (paired t tests) was unchanged between genotypes (mAHP ~Δ1.5mV, sAHP ~ Δ1mV, p>0.05 unpaired t test). **e** Example traces of isolated small-conductance Ca^{2+}-activated SK currents and full inhibition by d-tubocurarine (dTC, grey). **f** dTC-sensitive current amplitude (left) and charge (right) for each genotype (WT

n=8, HOM n=10 cells, 4 mice/genotype, p>0.05 unpaired t tests). **g** Example traces of spontaneous excitatory postsynaptic currents in WT and HOMS15A mice **h** Distribution of measured sEPSC properties for each genotype (WT: 1422-34 events, n=8 cells, HOM: 1455-65 events, n=7 cells; mean amp=14.0±0.8 *vs* 14.7±0.5 pA; instantaneous frequency=4.0±0.4 *vs* 4.3±0.3 Hz; N=3 mice/genotype; p>0.05, unpaired t tests). Data is presented as mean ± S.E.M or in box or violin plots representing minimum, maximum, median, 25/75 percentile and mean (indicated by a marker). Source data are provided as a Source Data file.

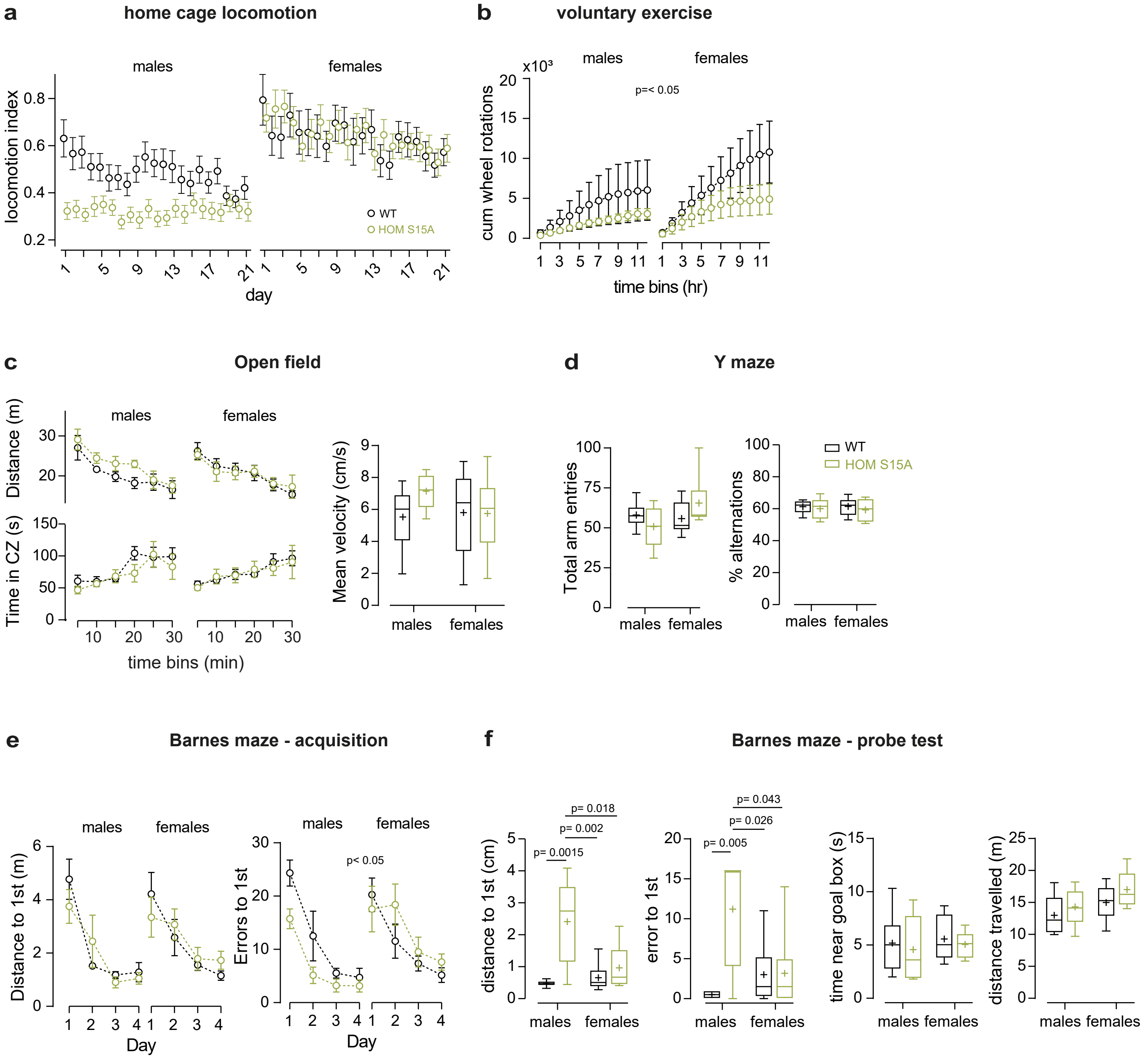

Supplementary Fig. 6: Additional behavioural characterization of Cav2.3 S15A mice. **a** Average home cage night-time locomotion of WT and HOM S15A mice (males WT n=5, HOM n=4; females WT n=4, HOM n=5) per day over a three-week period. **b** Cumulative wheel rotations for the same mouse cohort as **a**, based on the hourly average night time exercise over a two-week period (males WT n=5, HOM n=3; females WT n=4, HOM n=5; time x genotype p=0.035, Three-Way ANOVA). **c** left: Total distance travelled in an open field arena or time spent in the central zone (CZ) by WT (males n=6, females n=8) and HOM S15A (males n=5, females n=6) mice (p>0.05 Two-Way ANOVA,Tukey's); right: mean displacement velocity during the open field test (p>0.05). **d** Percentage of total arm entries (left) and successful spontaneous alternations (right) in the Y-maze test (p>0.05, Two-Way ANOVA, Tukey's). **e** Acquisition phase of the Barnes maze test with improved performance for both groups in the first 4 training days: distance travelled (left) and number of errors (right) during the 1st visit to the escape box (p>0.05, Two-Way ANOVA,

Sidak's), Errors to 1st: sex x genotype p=0.043, Three-Way ANOVA; for males p=0.08 on day 1, Two-Way ANOVA, Sidak's). **f** left: Memory assessment on probe day 5 (Two-Way ANOVA, Tukey's), for all measures: sex x genotype p=0.01, Three-Way ANOVA); right: Quantification of total time spent around the expected location of the goal box and total distance travelled on probe test day (p>0.05, Two-Way ANOVA, Tukey's). Data is presented as mean ± S.E.M or in box plots representing minimum, maximum, median, 25/75 percentile and mean (indicated by a marker). Source data are provided as a Source Data file.

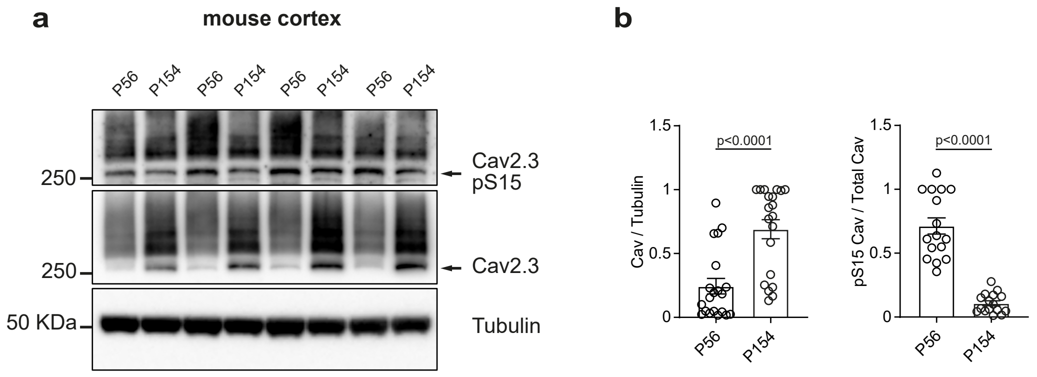

Supplementary Fig. 7: Relative phospho S15 Cav2.3 levels are reduced with age. a Representative Western Blot of male WT mouse cortical lysates at P56 (8 weeks) and P154 (22-23 weeks) showing that while total channel levels increase with age (middle blot), relative phosphorylation levels at S15 are reduced (top blot) **b** Corresponding quantification of total Cav (n=20, 4 animals/age; 5 technical replicates) and Cav pS15 (n=164 animals/age, 4 technical replicates); p<0.0001, two-tailed unpaired t test. Band intensity was normalized first to corresponding control (as indicated in the y axis) and then to maximal signal within each blot. Data is presented as mean ± S.E.M. Source data are provided as a Source Data file.

Supplementary References

1. Helbig KL*, et al.* De Novo Pathogenic Variants in CACNA1E Cause Developmental and Epileptic Encephalopathy with Contractures, Macrocephaly, and Dyskinesias. *American journal of human genetics* **103**, 666-678 (2018).

2. Ortiz Cabrera NV*, et al.* Dystonia and Contractures are Potential Early Signs of CACNA1E-Related Epileptic Encephalopathy. *Mol Syndromol* **12**, 25-32 (2021).

3. Royer-Bertrand B*, et al.* De novo variants in CACNA1E found in patients with intellectual disability, developmental regression and social cognition deficit but no seizures. *Mol Autism* **12**, 69 (2021).

4. Olson HE*, et al.* Cyclin-Dependent Kinase-Like 5 Deficiency Disorder: Clinical Review. *Pediatr Neurol* **97**, 18-25 (2019).

5. Rumenapp U*, et al.* The M3 muscarinic acetylcholine receptor expressed in HEK-293 cells signals to phospholipase D via G12 but not Gq-type G proteins: regulators of G proteins as tools to dissect pertussis toxin-resistant G proteins in receptor-effector coupling. *The Journal of biological chemistry* **276**, 2474-2479 (2001).
