# Supplementary material for: Epilepsy-linked kinase CDKL5 phosphorylates voltage-gated calcium channel Cav2.3, altering inactivation kinetics and neuronal excitability
Source: Nat Commun. 2023 Dec 11;14:7830. doi: 10.1038/s41467-023-43475-w (PMC10713615; doi:10.1038/s41467-023-43475-w)
Supplement: Supplementary file 3 — Reporting Summary [file 41467_2023_43475_MOESM3_ESM.pdf]

nature portfolio

Corresponding author(s): Marisol Sampedro-Castañeda
Sila K Ultanir

Last updated by author(s): Oct 25, 2023

Reporting Summary

Nature Portfolio wishes to improve the reproducibility of the work that we publish. This form provides structure for consistency and transparency in reporting. For further information on Nature Portfolio policies, see our Editorial Policies and the Editorial Policy Checklist.

Statistics

For all statistical analyses, confirm that the following items are present in the figure legend, table legend, main text, or Methods section.

n/a	Confirmed	
☐	☒	The exact sample size (*n*) for each experimental group/condition, given as a discrete number and unit of measurement
☐	☒	A statement on whether measurements were taken from distinct samples or whether the same sample was measured repeatedly
☐	☒	The statistical test(s) used AND whether they are one- or two-sided<br>*Only common tests should be described solely by name; describe more complex techniques in the Methods section.*
☐	☒	A description of all covariates tested
☐	☒	A description of any assumptions or corrections, such as tests of normality and adjustment for multiple comparisons
☐	☒	A full description of the statistical parameters including central tendency (e.g. means) or other basic estimates (e.g. regression coefficient) AND variation (e.g. standard deviation) or associated estimates of uncertainty (e.g. confidence intervals)
☐	☒	For null hypothesis testing, the test statistic (e.g. *F*, *t*, *r*) with confidence intervals, effect sizes, degrees of freedom and *P* value noted<br>*Give P values as exact values whenever suitable.*
☒	☐	For Bayesian analysis, information on the choice of priors and Markov chain Monte Carlo settings
☐	☒	For hierarchical and complex designs, identification of the appropriate level for tests and full reporting of outcomes
☒	☐	Estimates of effect sizes (e.g. Cohen's *d*, Pearson's *r*), indicating how they were calculated

Our web collection on statistics for biologists contains articles on many of the points above.

Software and code

Policy information about availability of computer code

Data collection	LTQ Orbitrap Velos mass spectrometer: elite software<br>Amersham 600 software<br>Zeiss 880 software - Zen 2<br>Open Source Instruments Software, Inc. (for wireless transmitter recordings of ECoG)<br>PClamp 10 (Molecular Devices)<br>Digital Ventilated Cage system (Techniplast) software<br>Automated rotarod - Ugo Basile model 47650
Data analysis	MaxQuant 105 (version 1.3.0.5)<br>Perseus (v.1.6.15.0)<br>Fiji 2.1<br>Clampfit 10.7/11.2<br>OriginPro 9.8<br>Semi-automated automated learning Python code - PyeCog - https://github.com/jcornford/pyecog (in: Colasante G, et al. In vivo CRISPRa decreases seizures and rescues cognitive deficits in a rodent model of epilepsy. Brain : a journal of neurology 143, 891-905 (2020)<br>DVC Analytics 3.4.0 (Techniplast)<br>Ethovision XT 15 (Noldus Information Technology)<br>G*Power 3.1 - https://www.psychologie.hhu.de/arbeitsgruppen/allgemeine-psychologie-und-arbeitspsychologie/gpower - (in: Faul, F., Erdfelder, E., Lang, A.-G., & Buchner, A. (2007). G*Power 3: A flexible statistical power analysis program for the social, behavioral, and biomedical sciences. Behavior Research Methods, 39, 175-191)

	GraphPad Prism 9

For manuscripts utilizing custom algorithms or software that are central to the research but not yet described in published literature, software must be made available to editors and reviewers. We strongly encourage code deposition in a community repository (e.g. GitHub). See the Nature Portfolio guidelines for submitting code & software for further information.

Data

Policy information about availability of data

All manuscripts must include a data availability statement. This statement should provide the following information, where applicable:

- Accession codes, unique identifiers, or web links for publicly available datasets
- A description of any restrictions on data availability
- For clinical datasets or third party data, please ensure that the statement adheres to our policy

- The quantitative proteomics dataset generated from WT and CDKL5 KO mouse cultures has been deposited to the ProteomeXchange Consortium via the PRIDE
- Peptide identification: Andromeda search engine against the Mus musculus canonical sequences downloaded from UniProtKB (Aug 2012, 77938 sequences)
- alpha1E subunit: accession - gene bank L27745.2
- The rest of the datasets analysed during this study are available from the corresponding authors on reasonable request

Research involving human participants, their data, or biological material

Policy information about studies with human participants or human data. See also policy information about sex, gender (identity/presentation), and sexual orientation and race, ethnicity and racism.

Reporting on sex and gender	N/A
Reporting on race, ethnicity, or other socially relevant groupings	N/A
Population characteristics	N/A
Recruitment	N/A
Ethics oversight	N/A

Note that full information on the approval of the study protocol must also be provided in the manuscript.

Field-specific reporting

Please select the one below that is the best fit for your research. If you are not sure, read the appropriate sections before making your selection.

☒ Life sciences ☐ Behavioural & social sciences ☐ Ecological, evolutionary & environmental sciences

For a reference copy of the document with all sections, see nature.com/documents/nr-reporting-summary-flat.pdf

Life sciences study design

All studies must disclose on these points even when the disclosure is negative.

Sample size	For mouse behaviour experiments we used the power analysis software G*Power tool (University of Dusseldorf) to estimate the minimum number of animals required to detect differences due to genotype and/or sex. This analysis was based on observations from previous behaviour experiments in a mixed cohort and yielded a >75% power for a cohort of 6 animals per gender/genotype. The statistical analysis of the behaviour dataset suggests that these experiments are sufficiently powered to detect differences due to various experimental factors.<br><br>For Western Blot and electrophysiology data, sample size was not predetermined. Instead, we analyzed the statistical differences between groups and based our conclusions on the probability of obtaining the observed effect sizes, with significance set at less tan 5%
Data exclusions	For electrophysiology analysis data exclusion criteria were established following preliminary experiments and were as follow:<br>For all HEK293 transfection conditions, cells with outward currents >40% of the peak current at +10mV or with inactivation tau that did not decay uniformly with depolarization were excluded from analysis. Similarly, cells were not used for current density or inactivation comparisons if run-down was >30% of the maximal current recorded in the experiment as this is known to affect inactivation time.<br>For voltage clamp recordings of R-type currents in brain slices a positive correlation was found between inactivation tau and Cm for Cm<80pF, thus this was established as the cut off cell size criteria for inclusion in inactivation tau comparisons. Cells with large cells outward currents at depolarising voltages (>50% of the peak current at +10mV) were also excluded.
Replication	Recombinant electrophysiology data were obtained from 2 HEK293 stable cell lines (expressing 2 different beta subunit) and two different experimenters. The results of these experiments were similar regardless of accessory beta subunit expressed. In order to minimise variability, recordings were performed from all experimental transfection conditions (construct combinations) on the same day in no particular order. Recordings were obtained from 3-8 independent transfections and recording days. HEK293 immunocytochemistry data were obttained from

	2 independent transfections.<br><br>All mouse data was collected and analysed blindly; littermates were used where possible. For Ca2+ current recordings in slices, both genotypes were recorded on the same day. Mouse electrophysiology data derive from 5 - 8 independent experiments<br><br>Western blot data has been replicated across different individuals from the same genotype: a minimum of two independent WB experiments were performed to evaluate changes in phosphoCav2.3 or total protein levels.
Randomization	Male and Female mice were allocated randomly to in vivo and in vitro experiments and analysis. In the case of behavioural data the numbers of animal/sex/genotype was pre-determined in order to investigate sex-dependent effects.<br>For HEK293 experiments multiple and if possible all transfection conditions were recorded on the same day to ensure that similar numbers of cells are recorded from each culture/ transfection batch. No other randomization was used.
Blinding	For mouse electrophysiology, behaviour and seizure studies, experimenters were blinded during data acquisition and analysis.<br>For HEK293 experiments experimenter was not blinded during acquisition or analysis. Multiple/ all conditions were recorded on the same day to minimize variability, cells were selected for recording based on their expression levels, and uniform analysis pipeline was applied to the recording data sets, as established in the field.

Reporting for specific materials, systems and methods

We require information from authors about some types of materials, experimental systems and methods used in many studies. Here, indicate whether each material, system or method listed is relevant to your study. If you are not sure if a list item applies to your research, read the appropriate section before selecting a response.

Materials & experimental systems

n/a	Involved in the study
☐	☒ Antibodies
☐	☒ Eukaryotic cell lines
☒	☐ Palaeontology and archaeology
☐	☒ Animals and other organisms
☒	☐ Clinical data
☒	☐ Dual use research of concern
☒	☐ Plants

Methods

n/a	Involved in the study
☒	☐ ChIP-seq
☒	☐ Flow cytometry
☒	☐ MRI-based neuroimaging

Antibodies

Antibodies used	Primary Antibodies:<br><br>WB<br>0. mouse Anti alpha tubulin (Sigma T9026) - clone DM1A<br>1. mouse Anti-HA.11 epitope tag Biolegend (901513) - clone 16B12<br>2. rabbit Anti pS15 Cav2.3 Covalab (custom)<br>3. mouse Anti Cav alpha1E Synaptic Systems (152411) - clone 62C10<br>4. rabbit Anti CDKL5 Atlas (HPA002847)<br>5. mouse Anti rabbit GAPDH Abcam (ab8245) - clone 6C5<br><br>Immunocytochemistry:<br>6. rat anti HA (Roche 11867423001 - clone 3F10 or (1)<br>7. mouse anti CDKL5 (Santa Cruz sc-376314) - clone D12<br>8. rat anti-Flag (Thermo Fisher MA1-142) - clone L5<br>9. rabbit anti M3 (Alomone AMR-006)<br>10. anti GFP sdAb FluoTag-X4 (NanoTag Biochecnologies N0304<br>11.chicken anti GFP (Aves GFP-1020)<br><br>Secondary Antibodies:<br>donkey anti rat Cy3 (Jackson 712-165-153)<br>donkey anti-mouse 647 (Jackson 115-605-003)<br>goat anti-rabbit 405 (Thermo Fisher A48254)<br>goat anti-chicken 488 (Thermo Fisher A32931)<br>donkey Anti rabbit HRP Jackson (711-035-152)<br>donkey Anti mouse HRP Jackson (715-035-151)
Validation	Primary antibodies:<br>0. mouse Anti alpha tubulin (Sigma T9026) - validated for WB by manufacturer and extensively used in the literature >1000 references; present study: single band at expected MW in mouse brain lysates, similar to previous data PMID 30266824<br>1. mouse Anti-HA epitope tag Biolegend (901513) - validation by manufacturer or in the literature: WB and immunofluorescence in cells lines expressing various tagged proteins; present study: bands at expected MW in WB for full length HA-Cav<br>2. rabbit Anti mouse pS15 Cav2.3 Covalab (custom) - validated for WB in current study using overexpression in HEK293 cells and

brain lysates from phosphomutant Cav2.3 mice where phosphoCav band is absent
3. mouse Anti rat Cav alpha1E Synaptic Systems (152411) - KO validated for WB: PMID 35045307; present study: band observed at expected MW in both mouse and human samples.
4. rabbit Anti human CDKL5 Atlas (HPA002847) - KO validated for WB: PMID 30266824
5. mouse Anti rabbit GAPDH Abcam (ab8245) - validated for WB by manufacturer and widely in the literature, preadsorption validated; single band of predicted MW observed in the present study
6. rat anti HA (Roche 11867423001): validated in the literatures and present study: low background fluorescence levels in untransfected cells, present study
7. mouse anti CDKL5 (Santa Cruz sc-376314): KO validated in the literature (WB: PMID 36613509) and present study: low background fluorescence in untransfected cells
8. rat anti-Flag (Thermo Fisher MA1-142) - single band at expected weight in WB for Flag-tagged protein, absent in untransfected cell lysates PMID 30266824
9. rabbit anti M3 (Alomone AMR-006): validated for IF by preadsorption in cortical tissue (manufacturer) and siRNA KO validation (PMID 25242372)
10. anti GFP sdAb FluoTag-X4 (NanoTag Biochecnologies N0304) - validated for IF by manufacturer using transfected GFP tagged protein in mammalian cells
11.chicken anti GFP (Aves GFP-1020) - extensively used in the literature (>2000 citations) and validated for IF using GFP-tagged protein or transgenic mouse tissue showing endogenous labelling of engineered cell types (see examples in manufacturers website); present study: GFP fluorescence as expected for membrane associated GFP-Dopamine receptor.

Secondary antibodies validated by manufacturers:
donkey Anti rabbit HRP Jackson (711-035-152)- affinity purified and tested by ELISA/adsorption, minimal crossreactivity with other species (10) serum proteins
donkey Anti mouse HRP Jackson (715-035-151)- affinity purified and tested by ELISA/adsorption, minimal crossreactivity with other species (10) serum proteins
donkey anti rat Cy3 (Jackson 712-165-153)- affinity purified and tested by ELISA and/or solid-phase adsorbed to ensure minimal cross-reaction with other species, but it may cross-react with immunoglobulins from other species.
donkey anti-mouse 647 (Jackson 115-605-003)-affinity purified and tested by ELISA and/or solid-phase adsorbed to ensure minimal cross-reaction with other species, but it may cross-react with immunoglobulins from other species.
goat anti-rabbit 405 (Thermo Fisher A48254)- highly cross-adsorbed antibody, IF validated in mammalian cells
goat anti-chicken 488 (Thermo Fisher A32931)-highly cross-adsorbed antibody, IF validated in mammalian cells

Eukaryotic cell lines

Policy information about cell lines and Sex and Gender in Research

Cell line source(s)	The HEK293 cell line stably expressing Cavβ3 and α2δ1 (HEK-a2d1-b3, inducible) was purchased from SB Drug discovery, Edinburgh, UK. HEK293 cells stably expressing β1b and α2δ1 were gifted by GSK. HEK293 cells, from Crick core facility.
Authentication	SB Drug Discovery validate their cell lines for gene integration and stable subunit expression by sequencing and in vitro electrophysiology (specific cell line authentication information was not provided). β1b and α2δ1 line we don't have information on what type of authentication they were subjected to.
Mycoplasma contamination	Regular mycoplasma testing is done in our cultures and these are negative.
Commonly misidentified lines (See ICLAC register)	No commonly misidentified cell lines were used in this study

Animals and other research organisms

Policy information about studies involving animals; ARRIVE guidelines recommended for reporting animal research, and Sex and Gender in Research

Laboratory animals	All mice are Mus musculus, C57BL6 genetic background (including Wild type animals, Cdkl5 knokcout colony and Cav2.3 phosphomutant mice). Ages used were post-embryonic day 16.5 to postnatal day 280
Wild animals	No wild animals were used in this study.
Reporting on sex	We reported individual sexes in our manuscript.
Field-collected samples	No field collected samples were used in this study.
Ethics oversight	The mice were bred and handled according to the Animals (Scientific Procedures) Act 1986 of the United Kingdom. Protocols and procedures were approved by The UK Home Office and institutional review bodies: The Francis Crick Institute ethical committee and the UCL Animal Welfare and Ethical Review Body.

Note that full information on the approval of the study protocol must also be provided in the manuscript.

Plants

Seed stocks	*Report on the source of all seed stocks or other plant material used. If applicable, state the seed stock centre and catalogue number. If plant specimens were collected from the field, describe the collection location, date and sampling procedures.*
Novel plant genotypes	*Describe the methods by which all novel plant genotypes were produced. This includes those generated by transgenic approaches, gene editing, chemical/radiation-based mutagenesis and hybridization. For transgenic lines, describe the transformation method, the number of independent lines analyzed and the generation upon which experiments were performed. For gene-edited lines, describe the editor used, the endogenous sequence targeted for editing, the targeting guide RNA sequence (if applicable) and how the editor was applied.*
Authentication	*Describe any authentication procedures for each seed stock used or novel genotype generated. Describe any experiments used to assess the effect of a mutation and, where applicable, how potential secondary effects (e.g. second site T-DNA insertions, mosiacism, off-target gene editing) were examined.*
